# From Adversity to Short Video Addiction: Psychological Distress and Online Social Support as Mediators Among Rural Chinese Left-Behind and Non-Left-Behind Adolescents

**DOI:** 10.3390/bs16091497

**Published:** 2026-08-26

**Authors:** Miao Chao, Qian Sun, Qi Liu, Yu Zhang, Xueling Weng

**Affiliations:** 1 Key Research Base of Humanities and Social Sciences of the Ministry of Education, Academy of Psychology and Behavior, Tianjin Normal University, Tianjin 300387, China; 2 Faculty of Psychology, Tianjin Normal University, Tianjin 300387, China; 3 Publicity and United Front Work Department (Faculty Affairs Office), Heilongjiang Polytechnic, Harbin 150080, China; 4 Student Mental Health Center, Guizhou College of Electronic Science and Technology, Guian 561113, China

**Keywords:** short video addiction, left-behind children, online social support, social anxiety, I-PACE model, adolescents

## Abstract

Short-form video platforms have become embedded in adolescents’ everyday lives, and rural left-behind children may be particularly vulnerable to problematic use in the context of prolonged parental absence. This cross-sectional study examined associations between family, peer, and school adversity and short video addiction (SVA), as well as whether these associations differed between left-behind children (LBC) and non-left-behind children (NLBC). Guided by selected components of the I-PACE model and ecological systems theory, we surveyed 1839 rural Chinese secondary school students, including 876 LBC and 963 NLBC. Bullying victimization, academic stress, parental rejection, stress, anxiety, social anxiety, online social support (OSS), and SVA were measured. Partial least squares structural equation modelling and multi-group analysis were used. LBC reported higher mean levels of bullying victimization, academic stress, stress, anxiety, social anxiety, and SVA than NLBC, although the corresponding effect sizes were small. Among LBC, significant indirect associations linked bullying victimization and academic stress with SVA through social anxiety, both independently and serially through OSS; academic stress was also indirectly associated with SVA through OSS alone. Among NLBC, significant indirect associations involving general stress and social anxiety linked adversity with SVA, whereas OSS alone was involved only in the academic stress–SVA association. No between-group path difference remained significant after Bonferroni or false discovery rate correction. The findings identify social anxiety and academic stress as relevant prevention targets but do not establish causal pathways or robust structural differences between LBC and NLBC.

## 1. Introduction

China’s rapid urbanization over the past three decades has produced large-scale internal migration, with millions of rural parents moving to cities for work while their children remain in home villages. These children, separated from at least one parent for six months or more, are referred to as left-behind children (LBC). Census-based estimates indicate that China had 66.93 million LBC in 2020, including 41.77 million rural LBC, who accounted for 37.9% of all rural children (National Bureau of Statistics of China et al., 2023). In rural China, parental labor migration can reorganize daily caregiving: children may remain with a non-migrant parent, grandparents, or other relatives, and the quality and stability of these arrangements can vary considerably (Fellmeth et al., 2018; Xie et al., 2023). Such intergenerational and kin-based care occurs within a cultural context in which extended family members often share child-rearing responsibilities, but it should not be assumed to be uniform across LBC. Prolonged parental absence has been associated with diminished parental warmth, peer victimization, academic pressures, and broader psychological distress (Fellmeth et al., 2018; Feng et al., 2024).

Short-form video platforms have become a prominent part of minors’ digital lives in China. According to the Fifth National Survey Report on Minors’ Internet Use, 54.1% of underage internet users regularly watched short videos in 2022, and 32.9% had filmed and posted short videos on platforms such as Douyin, Kuaishou, and WeChat in the previous year (Department for the Protection of Youth Rights and Interests of the Central Committee of the Communist Youth League & China Internet Network Information Center, 2023). Short video addiction (SVA) refers to compulsive engagement with short-form platforms that causes functional impairment and is difficult to control voluntarily (Liao, 2024). Growing evidence suggests that LBC are at higher risk than their peers. The absence of parents, limited supervision, and unmet social needs can create conditions in which digital platforms become a substitute for offline relationships and emotional support (Zhang & Chao, 2025).

The present study is guided by the I-PACE (Interaction of Person-Affect-Cognition-Execution) model developed by Brand et al. (2016, 2019). This model provides a framework for explaining how internet-use disorders develop and are maintained over time. It proposes that stable individual characteristics, such as personality traits and existing psychological symptoms, interact with affective and cognitive responses to stressful situations. These responses may be accompanied by craving and reduced inhibitory control, making repeated use of a particular application more likely and, over time, more habitual. The present study focuses on one selected component of this broader framework: the affect-related responses associated with adversity. Stress, anxiety, and social anxiety were therefore examined as potential statistical intermediaries between contextual adversity and SVA. The study does not constitute a full test of I-PACE because cognitive biases, craving, inhibitory control, and execution processes were not measured, and the cross-sectional design cannot evaluate the temporal process proposed by the model.

The choice of adversity variables in this study draws on Bronfenbrenner’s (1979) ecological systems theory. We use the theory as an organizing framework for locating adversity within the family, school, and peer microsystems, rather than assuming that developmental processes are culturally invariant. In the Chinese rural migration context, parental absence may alter the structure and meaning of the family microsystem, while examination-oriented schooling and peer relationships provide additional context-specific sources of strain. Within this framework, the present study selected one adversity indicator from each of these proximal contexts: parental rejection from the family context, academic stress from the school context, and bullying victimization from the peer context. Parental rejection, which refers to perceived parental hostility and emotional coldness, undermines children’s sense of security and capacity for emotional regulation, making them more vulnerable to psychological distress and compensatory internet use (Tom et al., 2023). Academic stress reflects the pressure that adolescents experience from examinations and homework. It has been linked to internalizing symptoms and excessive technology use among Chinese students (Liu & Lu, 2012; Wang et al., 2020). Bullying victimization covers physical, verbal, relational, and cyber forms of peer aggression. Adolescents who are victimized by peers show higher levels of anxiety, social withdrawal, and problematic media use (Jiang et al., 2024; Yao et al., 2025). By including adversity from all three primary social contexts, this study situates SVA risk within a broader ecological account of adolescent development.

Consistently with the I-PACE model, this study examines psychological distress as the affect-related component that mediates the link between stressor exposure and addictive behavior. Three aspects of distress are considered: general stress, anxiety, and social anxiety. General stress was selected to capture diffuse tension and irritability that may arise across family, school, and peer stressors. Anxiety was included to capture worry and physiological arousal, providing a broader internalizing response distinct from context-specific stress. Social anxiety was examined separately because fear of negative evaluation is directly relevant to interpersonal avoidance and the appeal of lower-risk online interaction. Together, the three dimensions represent complementary broad and interpersonal affective responses within the affect-related component of I-PACE. This rationale is also informed by compensatory internet use theory (CIUT), which proposes that individuals may turn to online activities to cope with offline distress or unmet psychosocial needs (Kardefelt-Winther, 2014). For socially anxious adolescents, online environments may provide interaction with lower perceived social risk than face-to-face settings (Lee & Stapinski, 2012; She et al., 2023). CIUT describes a possible coping process, however, and the present cross-sectional data cannot establish its temporal sequence.

A further pathway of interest involves online social support (OSS). OSS refers to emotional, informational, and companionate support that individuals perceive through online interactions. In many contexts, OSS is associated with positive wellbeing outcomes. At the same time, a meta-analysis of 26 studies involving more than 23,000 participants found a positive association between OSS and problematic internet use (Chao et al., 2025). Several explanations are possible: support-seeking may be associated with more frequent platform engagement, heavier users may have more opportunities to perceive online support, or both may reflect unmeasured social needs. The present study therefore examines OSS as a correlate within a theory-specified statistical model without assuming that OSS temporally precedes or increases SVA.

Whether the processes described above differ between LBC and NLBC is an open question. The developmental context of LBC, shaped by prolonged parental absence, may alter how rejection is experienced, how distress is managed, and how adversity translates into digital addiction. Comparative evidence is limited and findings have been inconsistent (Zhang & Chao, 2025). To address this gap, the present cross-sectional study used partial least squares structural equation modelling (PLS-SEM) and multi-group analysis (MGA) with 1839 rural secondary school students from three provinces in China. The objectives were to examine associations between adversity in family, peer, and school contexts and SVA; to estimate theory-specified indirect associations involving distress and OSS; and to assess whether the structural associations were comparable between LBC and NLBC. These models represent assumed statistical structures rather than demonstrated temporal pathways.

## 2. Method

### 2.1. Participants

Participants were recruited through cluster sampling from several township secondary schools in Shanxi, Yunnan, and Guangxi, China. Initially, 1997 students completed the survey. Because the measures referred specifically to experiences on short-video applications, respondents who reported no use of such applications were excluded from the present analyses. These excluded respondents were non-users, not a comparison group defined as being without SVA. Among the included users, SVA was analyzed as a continuous score, and no diagnostic cutoff was used to classify participants as addicted or non-addicted. The final sample comprised 1839 adolescents, including 1112 girls (60.5%), aged between 12 and 18 years (*M* = 15.96, *SD* = 1.68).

Among the participants, 876 (47.6%) were identified as left-behind children and 963 (52.4%) as non-left-behind children. Additional characteristics of the LBC group are presented in Table 1. Most LBC were from intact families (82.1%). Paternal grandparents were the most frequently reported caregivers (41.7%), followed by parents (29.6%), other caregivers (17.9%), other relatives (5.7%), and maternal grandparents (5.1%). Among adolescents with available responses, 58.6% reported that their fathers were currently away and 54.1% reported that their mothers were currently away. Weekly parent–child contact was reported by 54.7%, whereas 39.2% reported that parents returned home less than twice a year and 42.2% reported irregular return visits.

### 2.2. Data Collection Procedure

The survey was administered using paper-and-pencil questionnaires during mental health classes at the participating township secondary schools, with teachers distributing the questionnaires. Participation was voluntary and anonymous. Before completing the questionnaire, students were informed about the purpose of the study, confidentiality, and their right to withdraw at any stage without penalty. Informed consent and, for minor participants, consent or permission were obtained in accordance with the approved ethics protocol. A total of 1997 questionnaires were initially completed; respondents who reported no short-video application use were excluded from the platform-specific analyses, resulting in the final sample of 1839. The study protocol was reviewed and approved by the Ethics Committee of Tianjin Normal University (protocol code 2022012601).

### 2.3. Measures

#### 2.3.1. Left-Behind Status

Participants were asked whether one or both of their parents had worked outside their hometown for more than six months. Consistently with the definition commonly adopted in previous research, adolescents reporting prolonged parental migration were classified as left-behind children, whereas the remaining participants were categorized as non-left-behind children (He et al., 2012). Additional information regarding parental migration status, parent–child contact frequency, and parental return visits was collected from left-behind children.

#### 2.3.2. Bullying Victimization

Bullying victimization was measured using the general items of the revised Olweus Bully/Victim Questionnaire (Solberg & Olweus, 2003). The scale consists of four items measuring experiences of physical, verbal, social, and cyber bullying during the past few months. Responses were rated on a 5-point scale ranging from 1 (none) to 5 (several times per week), with higher scores indicating greater bullying victimization. Cronbach’s alpha in the present sample was 0.810.

#### 2.3.3. Academic Stress

Academic stress was measured using four items adapted from a previous study (Liu & Lu, 2012). Two items assessed “too much homework,” while the other two assessed “lack of academic achievement.” Each item was rated on a 4-point scale ranging from 1 (strongly disagree) to 4 (strongly agree). The four items were summed, with higher scores indicating greater academic stress. Cronbach’s alpha in the present sample was 0.826.

#### 2.3.4. Parental Rejection

Parental rejection was assessed using the six-item rejection dimension of the Chinese version of the 21-item short-form Egna Minnen Beträffande Uppfostran (s-EMBU) (Arrindell et al., 1999). Participants rated each item on a 4-point Likert scale ranging from 1 (Never) to 4 (Most of the time). Item scores were summed to obtain a total parental rejection score, with higher scores indicating greater perceived parental rejection. Cronbach’s alpha in the present sample was 0.842.

#### 2.3.5. Stress

Stress was measured using the seven-item Stress subscale of the Depression Anxiety Stress Scale-21 (DASS-21) (Lovibond & Lovibond, 1995). The items assess persistent tension, irritability, and difficulty relaxing during the past week. Participants rated each item on a 4-point Likert scale ranging from 0 (Did not apply to me at all) to 3 (Applied to me very much or most of the time). Higher scores indicated greater perceived stress. Cronbach’s alpha in the present sample was 0.873.

#### 2.3.6. Anxiety

Anxiety was measured using the seven-item Anxiety subscale of the Depression Anxiety Stress Scale-21 (DASS-21) (Lovibond & Lovibond, 1995). The items assess anxiety symptoms experienced during the past week. Participants rated each item on a 4-point Likert scale ranging from 0 (Did not apply to me at all) to 3 (Applied to me very much or most of the time). Higher scores indicated greater anxiety. Cronbach’s alpha in the present sample was 0.866.

#### 2.3.7. Social Anxiety

Social anxiety was assessed using the six-item Social Interaction Anxiety Scale (SIAS-6) (Peters et al., 2012). Each item was rated on a 5-point Likert scale from 1 (Not at all characteristic or true of me) to 5 (Extremely characteristic or true of me). Higher scores indicated greater social anxiety. Cronbach’s alpha in the present sample was 0.855.

#### 2.3.8. Online Social Support

Online social support was measured using an adapted four-item scale developed by Frison and Eggermont (2016). References to “Facebook” in the original items were replaced with “short video applications” to fit the present context (e.g., “I can find help on short video applications”). Each item was rated on a 5-point Likert scale ranging from 1 (Strongly Disagree) to 5 (Strongly Agree), with higher scores indicating stronger perceived online social support. Cronbach’s alpha in the present sample was 0.829.

#### 2.3.9. Short Video Addiction

Short video addiction was measured using a ten-item version adapted from the Smartphone Addiction Scale (SAS) (Kwon et al., 2013). Following previous research, references to “smartphone” were replaced with “short video” to focus the items on short-video platforms (Chao et al., 2023). Each item was rated on a 6-point Likert scale from 1 (Strongly Disagree) to 6 (Strongly Agree), with higher scores indicating more severe problematic short-video use. Cronbach’s alpha in the present sample was 0.933. The score was used as a continuous indicator and was not treated as a clinical diagnosis.

### 2.4. Data Analysis

Data analysis was conducted using IBM SPSS Statistics version 27 and SmartPLS 4. First, SPSS was used to calculate descriptive statistics, including means and standard deviations. Independent-samples *t*-tests were then conducted to compare mean scores between left-behind and non-left-behind adolescents.

Subsequently, partial least squares structural equation modeling (PLS-SEM) was conducted using SmartPLS to estimate the theory-specified associations among the constructs. Prior to examining the structural model, reliability and validity were evaluated. Measurement invariance was assessed using the MICOM procedure before multi-group analysis (PLS-MGA). Because multiple path differences were examined in the MGA, both Bonferroni and false discovery rate corrections were applied to the MGA *p* values. Additional confirmatory factor analyses were conducted for the two adapted measures, and an exploratory moderation analysis examined whether the associations varied by gender. Model fit was evaluated using the standardized root mean square residual (SRMR), with values below 0.10 treated as acceptable. Because all variables were measured concurrently, path directions and indirect effects were interpreted as statistical associations rather than evidence of temporal or causal ordering.

## 3. Results

### 3.1. Descriptive Statistics

Table 2 presents the descriptive statistics for the study variables across left-behind and non-left-behind adolescents. The final sample consisted of 1839 adolescents, including 876 left-behind children (47.6%) and 963 non-left-behind children (52.4%).

As shown in Table 2, LBC reported higher mean scores for bullying victimization, academic stress, stress, anxiety, social anxiety, and SVA than NLBC, whereas parental rejection and OSS did not differ significantly. Cohen’s d values ranged from 0.05 to 0.18; even the statistically significant group differences were small in magnitude (d = 0.09–0.18). The subsequent analyses therefore emphasize the pattern and magnitude of associations rather than treating small mean differences as evidence of broad group vulnerability.

### 3.2. Measurement Model

Prior to testing the structural model, the measurement model was assessed for reliability and validity. As shown in Table 3, all indicator loadings exceeded 0.50. Cronbach’s alpha ranged from 0.810 to 0.933, composite reliability ranged from 0.874 to 0.943, and average variance extracted ranged from 0.558 to 0.662. The HTMT values in Table 4 were below 0.85, supporting discriminant validity, and the SRMR was 0.087. Additional one-factor confirmatory factor analyses supported the expected structures of the adapted measures. The SVA scale showed acceptable fit (RMSEA = 0.084, 90% CI [0.080, 0.089], CFI = 0.935, TLI = 0.911, SRMR = 0.057), and the OSS scale showed good fit (RMSEA = 0.079, CFI = 0.986, TLI = 0.975, SRMR = 0.011). These results provide evidence for internal construct structure but do not replace validation against external criteria or behavioral indicators.

Discriminant validity was examined using the heterotrait–monotrait ratio of correlations (HTMT). As shown in Table 4, all HTMT values were below the recommended threshold of 0.85, supporting discriminant validity among the constructs. In addition, the standardized root mean square residual (SRMR) was 0.087, which was below the threshold of 0.10, indicating an acceptable model fit.

### 3.3. Structural Model for Left-Behind Children

**Figure 1:**
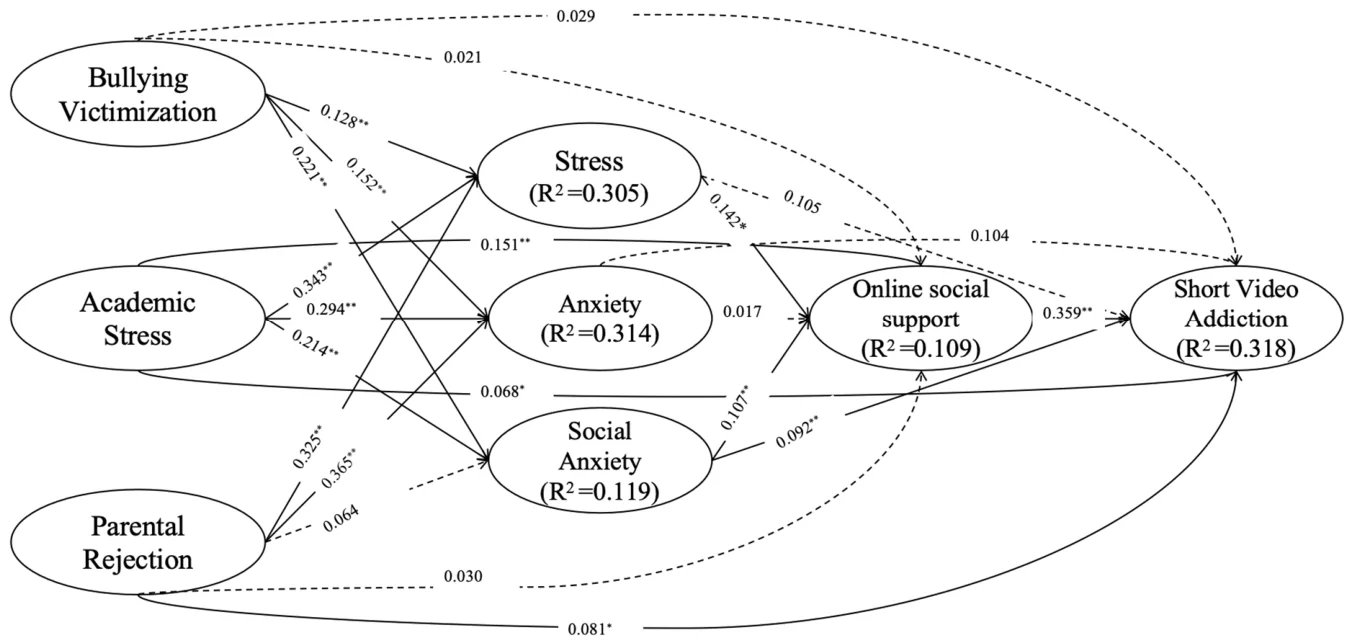
PLS results of the structural model for left-behind children. Solid paths are statistically significant and dashed paths are non-significant; * *p* < 0.05, ** *p* < 0.01.

The structural model was evaluated using the bootstrapping procedure in PLS-SEM. The R^2^ values were 0.305 for stress, 0.314 for anxiety, 0.119 for social anxiety, 0.109 for OSS, and 0.318 for SVA. As shown in Figure 1, bullying victimization, academic stress, and parental rejection showed statistically significant associations with at least one dimension of psychological distress among left-behind children. Bullying victimization was positively associated with stress (β = 0.128, *p* < 0.01), anxiety (β = 0.152, *p* < 0.01), and social anxiety (β = 0.221, *p* < 0.01).

Academic stress was positively associated with stress (β = 0.343, *p* < 0.01), anxiety (β = 0.294, *p* < 0.01), and social anxiety (β = 0.214, *p* < 0.01). The academic stress–stress coefficient was the largest of these three associations.

Parental rejection was positively associated with stress (β = 0.325, *p* < 0.01) and anxiety (β = 0.365, *p* < 0.01), but not with social anxiety. A positive direct association between parental rejection and SVA was also observed (β = 0.081, *p* < 0.05).

Stress (β = 0.142, *p* < 0.05) and social anxiety (β = 0.107, *p* < 0.01) were positively associated with OSS, whereas anxiety was not (β = 0.017, *p* > 0.05). Thus, higher stress and social anxiety scores were concurrently associated with greater perceived online social support among left-behind children.

OSS was positively associated with SVA. This concurrent association is compatible with several directions: support-seeking may accompany more frequent platform engagement, heavier users may perceive more support, or both may reflect unmeasured needs. It should therefore not be interpreted as evidence that OSS increases SVA risk. Among the distress variables, social anxiety showed statistically significant indirect associations linking bullying victimization and academic stress with SVA.

As shown in Table 5, statistically significant indirect associations through social anxiety were observed for bullying victimization and SVA (β = 0.020, *t* = 2.299, *p* = 0.022) and for academic stress and SVA (β = 0.020, *t* = 2.372, *p* = 0.018). The corresponding indirect associations through anxiety and general stress were not statistically significant (*p* > 0.05).

Within the theory-specified ordering, indirect associations through social anxiety and OSS were statistically significant for bullying victimization (β = 0.009, *t* = 2.298, *p* = 0.022) and academic stress (β = 0.008, *t* = 2.266, *p* = 0.024), but not for parental rejection. An indirect association through OSS was also observed between academic stress and SVA (β = 0.054, *t* = 3.907, *p* < 0.001). These estimates do not demonstrate that social anxiety or OSS temporally preceded SVA.

### 3.4. Structural Model for Non-Left-Behind Children

**Figure 2:**
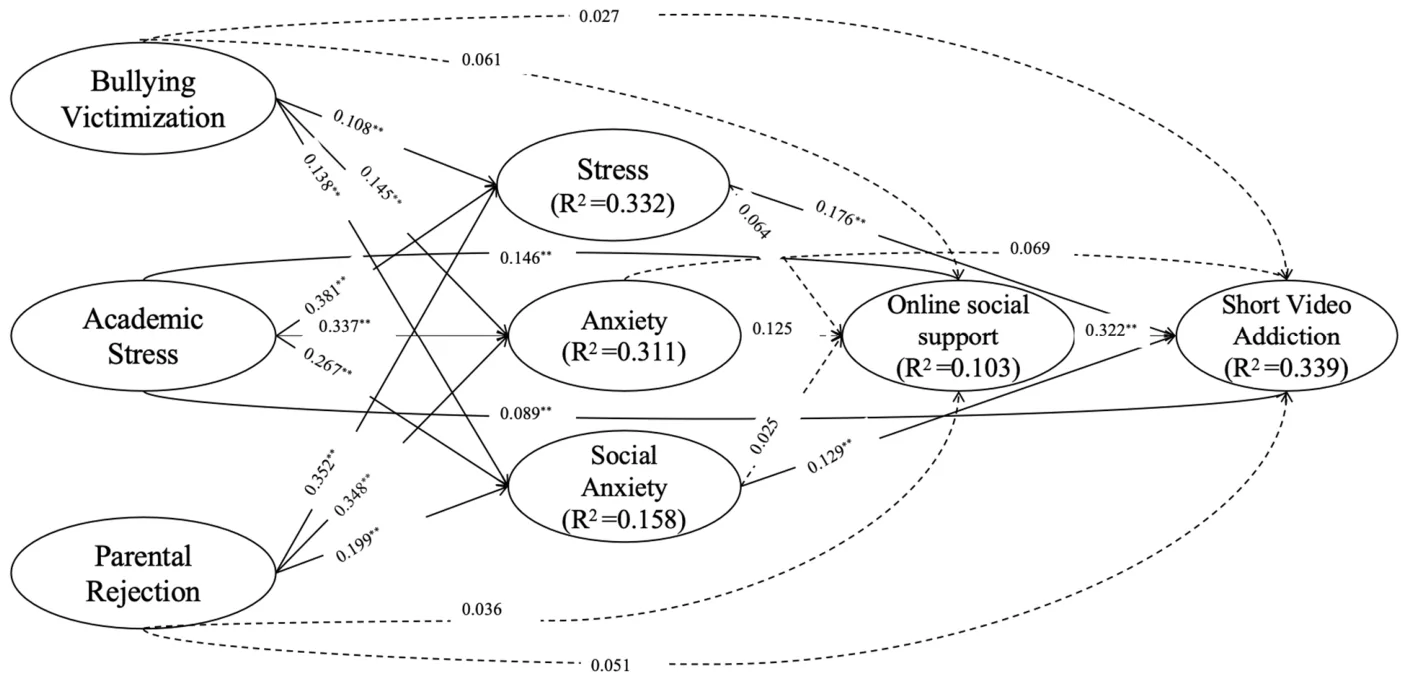
PLS results of the structural model for non-left-behind children. Solid paths are statistically significant and dashed paths are non-significant; ** *p* < 0.01.

As shown in Figure 2, the R^2^ values were 0.332 for stress, 0.311 for anxiety, 0.158 for social anxiety, 0.103 for OSS, and 0.339 for SVA. Academic stress, bullying victimization, and parental rejection were associated with psychological distress among non-left-behind children. Academic stress was positively associated with anxiety (β = 0.337, *p* < 0.001), social anxiety (β = 0.267, *p* < 0.001), stress (β = 0.381, *p* < 0.001), and SVA (β = 0.089, *p* = 0.004). Bullying victimization was positively associated with anxiety (β = 0.145, *p* < 0.001), social anxiety (β = 0.138, *p* < 0.001), and stress (β = 0.108, *p* < 0.001), whereas its direct association with SVA was not statistically significant (*p* = 0.377). Parental rejection was positively associated with anxiety (β = 0.348, *p* < 0.001), social anxiety (β = 0.199, *p* < 0.001), and stress (β = 0.352, *p* < 0.001), whereas its direct association with SVA was not statistically significant (*p* = 0.145).

Stress (β = 0.176, *p* = 0.003) and social anxiety (β = 0.129, *p* < 0.001) were positively associated with SVA, whereas anxiety was not (*p* > 0.05). Academic stress was positively associated with anxiety, social anxiety, stress, and OSS.

As shown in Table 6, several statistically significant indirect associations through stress and social anxiety were observed among non-left-behind children. The only statistically significant indirect association through OSS alone linked academic stress with SVA.

Bullying victimization showed statistically significant indirect associations with SVA through stress (β = 0.019, *p* = 0.022) and social anxiety (β = 0.018, *p* = 0.004). Academic stress showed indirect associations through stress (β = 0.067, *p* = 0.003), social anxiety (β = 0.034, *p* = 0.001), and OSS (β = 0.047, *p* < 0.001). Parental rejection showed indirect associations through stress (β = 0.062, *p* = 0.004) and social anxiety (β = 0.026, *p* = 0.002). Indirect associations through anxiety and most theory-specified serial paths involving OSS were not statistically significant. The indirect association from parental rejection through OSS to SVA was not statistically significant in either LBC (*p* = 0.457) or NLBC (*p* = 0.340).

### 3.5. Multi-Group Analysis

A multi-group analysis (PLS-MGA) was conducted after measurement invariance was evaluated using MICOM. Step 2 supported compositional invariance for all constructs because each original correlation exceeded its 5% permutation quantile. Step 3 did not establish equality of all composite means and variances; accordingly, partial rather than full measurement invariance was concluded. Full MICOM results are provided in Supplementary Table S1. The predominant MGA finding was similarity: most structural associations did not differ between LBC and NLBC. At the unadjusted threshold, the parental rejection–social anxiety path differed between groups (coefficient difference = −0.135, *p* = 0.008), with a stronger association among NLBC. However, this difference did not remain significant after either Bonferroni or false discovery rate correction, and no between-group path difference was statistically significant after correction. An additional moderation analysis found no statistically significant moderation by gender for the examined associations (all *p* > 0.05).

## 4. Discussion

This cross-sectional study examined theory-specified associations among adversity in family, peer, and school contexts, psychological distress, OSS, and SVA among rural Chinese adolescents. The I-PACE model informed the affect-related portion of the model, while ecological systems theory organized the contextual sources of adversity. The results should be understood as associational and do not establish temporal pathways. After correction for multiple MGA comparisons, the central between-group finding was the absence of robust structural differences.

### 4.1. Adversity Across Ecological Contexts and Psychological Distress

Consistently with the affect-related component of I-PACE, each adversity variable—peer victimization, academic stress, and parental rejection—was associated with at least one dimension of psychological distress in both groups. This is broadly consistent with prior literature showing that family, school, and peer adversity each contribute to internalizing symptoms among Chinese adolescents (Xiao et al., 2017; Moore et al., 2017; Feng et al., 2024). Academic stress showed strong and consistent associations with psychological distress and was involved in several statistically significant indirect associations with SVA, but the cross-sectional results do not establish it as a causal driver. Parental rejection was associated with stress and anxiety in both groups and with social anxiety in NLBC at the within-group level. The unadjusted MGA difference for the parental rejection–social anxiety path did not survive multiplicity correction, so the data do not support a reliable group-specific interpretation of that association. Ecological systems theory is used here as a context-sensitive organizing framework rather than evidence that the same processes operate identically across cultures. The study also tests only selected I-PACE components; cognition, craving, inhibitory control, and execution processes were not assessed.

Academic stress showed strong and consistent associations with psychological distress across both groups, including the largest coefficient for general stress. Parental rejection showed the largest association with anxiety in both groups. This is consistent with the central role of academic competition in the Chinese secondary education system, where examination pressures are a persistent source of daily strain for adolescents (Liu & Lu, 2012; Wang et al., 2020; Zhou et al., 2023). Academic stress was also involved in several statistically significant indirect associations with SVA, but the cross-sectional results do not establish academic pressure as a causal driver.

Within-group estimates showed that parental rejection was associated with stress and anxiety in both groups and with social anxiety only among NLBC. However, the unadjusted between-group difference for the parental rejection–social anxiety path did not survive Bonferroni or false discovery rate correction. This pattern is therefore not interpreted as evidence of adaptation or differential vulnerability.

The LBC model contained a statistically significant direct association between parental rejection and SVA, whereas the corresponding within-group coefficient was not significant in the NLBC model. The corresponding MGA comparison was not statistically significant, so the difference in within-group significance should not be interpreted as evidence that this path differs between LBC and NLBC. Within the LBC model, the association may reflect coping with unmet psychosocial needs or differences in parental monitoring (Kardefelt-Winther, 2014; Lukavská et al., 2020), but these mechanisms were not assessed and remain hypotheses for longitudinal research.

### 4.2. The Role of Social Anxiety as a Mediator

Social anxiety was involved in the most consistent indirect associations in the theory-specified models. Among LBC, significant indirect associations through social anxiety linked bullying victimization and academic stress with SVA. Among NLBC, indirect associations through social anxiety were observed for bullying victimization, academic stress, and parental rejection, while general stress was also involved in these associations. This pattern is consistent with the I-PACE proposition that affective responses may be relevant to problematic application use and with evidence that online environments can offer socially anxious adolescents participation with lower perceived risk of negative evaluation (Brand et al., 2019; Jiang et al., 2024). Jiang et al. (2024) found a similar chain mediation in which bullying victimization elevated social anxiety, which in turn predicted greater short video use. Nevertheless, concurrent measurement prevents conclusions about whether social anxiety preceded SVA.

The specific prominence of social anxiety among LBC may reflect a characteristic of this population’s developmental history. However, the present survey did not directly measure separation history, caregiver stability, or sensitivity to social evaluation. This interpretation is therefore offered as a hypothesis for future longitudinal research rather than an explanation established by the current data.

### 4.3. Online Social Support and Short Video Addiction

OSS is typically understood as a protective factor that buffers the psychological effects of stress. It is associated with higher life satisfaction and better mental health in many populations (Frison & Eggermont, 2016). In the present model, OSS was positively associated with SVA and was involved in indirect associations linking academic stress with SVA in both groups. Among LBC, theory-specified indirect paths involving social anxiety and OSS were also statistically significant for bullying victimization and academic stress. These findings establish concurrent associations only. They do not show that OSS amplified risk, because heavier short-video use may itself increase opportunities to perceive support, and unmeasured social needs may contribute to both OSS and SVA.

CIUT offers one possible interpretation of these associations: adolescents experiencing social anxiety or academic pressure may use online activities to cope with unmet needs, and perceived support may accompany repeated engagement (Kardefelt-Winther, 2014). An alternative interpretation is that frequent users encounter more interactions and therefore report greater OSS. Reciprocal influence is also possible. Longitudinal data are required to determine whether support-seeking precedes heavier use, results from it, or develops alongside it.

The theory-specified serial associations through social anxiety and OSS reached significance in the LBC model but not in the NLBC model. This within-group pattern does not by itself establish a significant difference between groups, nor does it demonstrate different support-seeking motives. Interpersonal coping among LBC and academically focused coping among NLBC are possible interpretations, but motives were not measured and these explanations remain tentative.

### 4.4. Between-Group Differences

The MGA results indicated that most structural pathways did not differ significantly between LBC and NLBC, suggesting broad similarity in the associations linking adversity, distress, OSS, and SVA. At the unadjusted threshold, parental rejection was more strongly associated with social anxiety among NLBC; however, this coefficient difference did not remain significant after Bonferroni or false discovery rate correction. The study therefore provides no robust evidence that the structural associations differ between groups, and the unadjusted coefficient is not used to infer adaptation or differential vulnerability.

### 4.5. Practical Implications

The findings suggest several practical directions. School-based prevention programmes combining skills training and media literacy have reduced problematic internet use among adolescents and young people in intervention research (Romero Saletti et al., 2021). Programmes could therefore strengthen coping, emotion regulation, self-regulation, and offline social skills while helping adolescents recognize problematic short-video use and develop balanced media routines. Given that rural schools in China often have limited access to professional mental health services, group-based programmes targeting negative social cognitions and avoidance behaviors could be delivered by teachers or school counsellors with appropriate training. In light of evidence linking academic stress and the school context with adolescent distress (Liu & Lu, 2012; Zhou et al., 2023), schools could consider academic-support resources that reduce avoidable examination and homework pressure. Parent education could emphasize supportive communication, realistic expectations, and responses that do not intensify rejection or conflict. These recommendations are research-informed proposals rather than interventions tested in the present study.

### 4.6. Limitations and Future Directions

Several limitations should be noted. First, the cross-sectional design does not permit causal inference or establish the direction and timing of the theory-specified indirect associations. Reverse and reciprocal relationships remain plausible; longitudinal evidence has shown bidirectional links between compulsive internet use and parent–child communication (van den Eijnden et al., 2010), and the concurrent association between OSS and SVA cannot determine whether perceived support precedes or follows repeated platform use. Longitudinal and cross-lagged studies are needed. Second, the sample came from rural secondary schools in Shanxi, Yunnan, and Guangxi and included a higher proportion of girls (60.5%), which limits generalizability. Although the exploratory analysis detected no significant moderation by gender, this null result does not establish equivalence across genders and should be examined in more balanced samples. Third, family structure, caregiver category, parental migration, contact frequency, and return visits were recorded descriptively for LBC, but their categorical response formats—including irregular and not-applicable categories—limited their use as covariates. Boarding status, caregiver quality, and mutually exclusive one-parent versus two-parent migration groups were not directly established, so meaningful heterogeneity remains within the broad LBC category. Fourth, all measures were self-reported. The additional CFAs supported the internal one-factor structures of the adapted OSS and SVA measures, but validation against behavioral use indicators and external criteria is still needed. Finally, the unadjusted parental rejection–social anxiety group difference did not survive correction for multiple comparisons, and the study should not be read as establishing differential structural vulnerability between LBC and NLBC.

## 5. Conclusions

Guided by selected components of I-PACE and ecological systems theory, this cross-sectional study identified associations among adversity, psychological distress, OSS, and SVA in rural Chinese adolescents. Theory-specified indirect associations involving social anxiety, general stress, and OSS varied across the two within-group models, but most structural associations were similar between LBC and NLBC. No between-group path difference remained statistically significant after Bonferroni or false discovery rate correction. The findings identify social anxiety, academic stress, and problematic media-use patterns as relevant topics for prevention research, while longitudinal studies are required before causal, temporal, or group-specific conclusions can be drawn.

## Figures and Tables

**Table 1:** Characteristics of left-behind children (n = 876).

Variables	n	Percent
Gender		
Female	501	57.2%
Male	375	42.8%
Family structure		
Intact family	717	82.1%
Single-parent family	101	11.6%
Stepfamily	55	6.3%
Caregiver		
Parents	258	29.6%
Maternal grandparents	44	5.1%
Paternal grandparents	363	41.7%
Other relatives	50	5.7%
Others	156	17.9%
Parental migration status		
Father currently away	497	58.6%
Mother currently away	449	54.1%
Father previously away	278	32.8%
Mother previously away	252	30.4%
Father never migrated	73	8.6%
Mother never migrated	129	15.5%
Frequency of parent–child contact		
Once a week	463	54.7%
Once every two weeks	46	5.4%
Once a month	21	2.5%
Once every two months	2	0.2%
Less than every two months	14	1.7%
Irregular contact	245	28.9%
Not applicable	56	6.6%
Frequency of parents visiting home		
Once a month	43	5.1%
Every 1–3 months	44	5.2%
Every 4–6 months	70	8.3%
Less than twice a year	332	39.2%
Irregular	357	42.2%

**Table 2:** Comparison of means for key variables between LBC and NLBC (N = 1839).

Variables	LBC (n = 876)		NLBC (n = 963)		*t*	*p*-Value	Cohen’s d
	*M*	*SD*	*M*	*SD*			
Bullying victimization	5.19	2.31	4.82	2.03	3.64	<0.001	0.17
Academic stress	13.07	3.71	12.61	3.85	2.58	0.010	0.12
Parental rejection	9.83	3.31	9.64	3.35	1.21	0.225	0.05
Stress	7.76	4.38	6.93	4.42	4.01	<0.001	0.18
Anxiety	7.08	4.35	6.30	4.43	3.79	<0.001	0.17
Social anxiety	14.80	5.51	14.28	5.70	1.97	0.049	0.09
Online social support	10.74	3.79	10.54	3.78	1.09	0.273	0.05
Short video addiction	28.18	11.24	26.33	11.37	3.49	<0.001	0.16

**Table 3:** Measurement model results.

Construct/Item	Mean	Standard Deviation	Standard Loading	Cronbach’s Alpha	CR	AVE
**Bullying Victimization (BV)**				0.810	0.874	0.634
BV1	1.15	0.56	0.735			
BV2	1.49	0.95	0.818			
BV3	1.20	0.64	0.856			
BV4	1.14	0.55	0.771			
**Academic Stress (AS)**				0.826	0.885	0.658
AS1	2.98	1.19	0.792			
AS2	3.31	1.17	0.741			
AS3	3.33	1.15	0.864			
AS4	3.21	1.17	0.842			
**Parental Rejection (PR)**				0.842	0.883	0.558
PPR1	1.63	0.72	0.746			
PPR2	1.55	0.73	0.741			
PPR3	1.85	0.83	0.749			
PPR4	1.49	0.72	0.785			
PPR5	1.54	0.72	0.768			
PPR6	1.67	0.74	0.689			
**Stress (STR)**				0.873	0.902	0.570
STR1	1.27	0.79	0.722			
STR2	0.95	0.80	0.756			
STR3	1.17	0.85	0.789			
STR4	1.03	0.86	0.809			
STR5	1.05	0.86	0.824			
STR6	0.95	0.79	0.634			
STR7	1.02	0.89	0.734			
**Anxiety (ANX)**				0.866	0.898	0.559
ANX1	1.01	0.78	0.648			
ANX2	0.78	0.80	0.741			
ANX3	0.82	0.83	0.727			
ANX4	1.43	0.95	0.647			
ANX5	0.88	0.83	0.809			
ANX6	0.84	0.85	0.820			
ANX7	0.84	0.88	0.818			
**Social Anxiety (SA)**				0.855	0.892	0.580
SA1	2.35	1.18	0.707			
SA2	2.24	1.14	0.783			
SA3	2.36	1.29	0.775			
SA4	2.61	1.33	0.773			
SA5	2.33	1.20	0.811			
SA6	2.62	1.23	0.716			
**Online Social Support (OSS)**				0.829	0.887	0.662
OSS1	3.05	1.14	0.785			
OSS2	2.86	1.18	0.857			
OSS3	2.53	1.19	0.837			
OSS4	2.18	1.15	0.773			
**Short Video Addiction (SVA)**				0.933	0.943	0.625
SVA1	2.98	1.46	0.733			
SVA2	2.93	1.45	0.794			
SVA3	2.78	1.48	0.728			
SVA4	2.48	1.42	0.807			
SVA5	2.35	1.35	0.821			
SVA6	2.63	1.40	0.849			
SVA7	2.49	1.37	0.838			
SVA8	2.57	1.43	0.771			
SVA9	3.10	1.52	0.784			
SVA10	2.85	1.50	0.773			

**Table 4:** HTMT discriminant validity results.

Construct	BV	AS	PR	STR	ANX	SA	OSS	SVA
BV	-							
AS	0.076	-						
PR	0.341	0.144	-					
STR	0.270	0.480	0.482	-				
ANX	0.305	0.438	0.507	0.718	-			
SA	0.270	0.318	0.249	0.457	0.468	-		
OSS	0.141	0.292	0.177	0.319	0.312	0.468	-	
SVA	0.201	0.333	0.282	0.466	0.457	0.350	0.511	-

Abbreviations: BV = Bullying Victimization, AS = Academic Stress, PR = Parental Rejection, STR = Stress, ANX = Anxiety, SA = Social Anxiety, OSS = Online Social Support, SVA = Short Video Addiction.

**Table 5:** Results of indirect association testing for left-behind children.

Path	β	*t*-Value	*p*-Value
BV → STR → SVA	0.013	1.540	0.124
BV → ANX → SVA	0.016	1.558	0.119
BV → SA → SVA	0.020	2.299	0.022
BV → STR → OSS → SVA	0.006	1.709	0.088
BV → ANX → OSS → SVA	0.001	0.249	0.804
BV → SA → OSS → SVA	0.009	2.298	0.022
BV → OSS → SVA	0.007	0.607	0.544
AS → STR → SVA	0.036	1.751	0.080
AS → ANX → SVA	0.030	1.683	0.093
AS → SA → SVA	0.020	2.372	0.018
AS → STR → OSS → SVA	0.017	1.949	0.051
AS → ANX → OSS → SVA	0.002	0.259	0.796
AS → SA → OSS → SVA	0.008	2.266	0.024
AS → OSS → SVA	0.054	3.907	<0.001
PR → STR → SVA	0.034	1.726	0.084
PR → ANX → SVA	0.038	1.664	0.096
PR → SA → SVA	0.006	1.338	0.181
PR → STR → OSS → SVA	0.017	1.920	0.055
PR → ANX → OSS → SVA	0.002	0.258	0.797
PR → SA → OSS → SVA	0.002	1.341	0.180
PR → OSS → SVA	0.011	0.743	0.457

Abbreviations: BV = Bullying Victimization, AS = Academic Stress, PR = Parental Rejection, STR = Stress, ANX = Anxiety, SA = Social Anxiety, OSS = Online Social Support, SVA = Short Video Addiction.

**Table 6:** Results of indirect association testing for non-left-behind children.

Path	β	*t*-Value	*p*-Value
BV → STR → SVA	0.019	2.283	0.022
BV → ANX → SVA	0.010	1.13	0.258
BV → SA → SVA	0.018	2.863	0.004
BV → STR → OSS → SVA	0.002	0.872	0.383
BV → ANX → OSS → SVA	0.006	1.652	0.099
BV → SA → OSS → SVA	0.001	0.619	0.536
BV → OSS → SVA	0.020	1.537	0.124
AS → STR → SVA	0.067	2.930	0.003
AS → ANX → SVA	0.023	1.190	0.234
AS → SA → SVA	0.034	3.377	0.001
AS → STR → OSS → SVA	0.008	0.920	0.358
AS → ANX → OSS → SVA	0.014	1.821	0.069
AS → SA → OSS → SVA	0.002	0.643	0.520
AS → OSS → SVA	0.047	3.633	<0.001
PR → STR → SVA	0.062	2.896	0.004
PR → ANX → SVA	0.024	1.176	0.240
PR → SA → SVA	0.026	3.106	0.002
PR → STR → OSS → SVA	0.007	0.917	0.359
PR → ANX → OSS → SVA	0.014	1.801	0.072
PR → SA → OSS → SVA	0.005	0.631	0.528
PR → OSS → SVA	0.012	0.950	0.340

## Data Availability

The data presented in this study are available on request from the corresponding author due to privacy and ethical restrictions designed to protect minor participants and the confidentiality of the sensitive psychosocial and family information collected.

## Supplementary Materials

The following supporting information can be downloaded at: https://www.mdpi.com/article/10.3390/bs16091497/s1, Table S1: Measurement invariance of composites (MICOM).
