# Supplementary material for: From Adversity to Short Video Addiction: Psychological Distress and Online Social Support as Mediators Among Rural Chinese Left-Behind and Non-Left-Behind Adolescents
Source: Behav Sci (Basel). 2026 Aug 26;16(9):1497. doi: 10.3390/bs16091497 (PMC13603289; doi:10.3390/bs16091497)
Supplement: Supplementary file 1 [file behavsci-16-01497-s001.zip › behavsci-4433131-supplementary.pdf]

Supplementary Table S1. Measurement invariance of composites (MICOM)

Step 2: Compositional Invariance

Construct	Original correlation	5% Quantile	Permutation p-value
BV	0.996	0.995	**0.081**
AS	1.000	0.999	0.494
PR	0.999	0.998	**0.542**
STR	1.000	0.999	0.138
ANX	0.999	0.999	**0.112**
SA	1.000	0.998	0.603
OSS	1.000	0.998	0.368
SVA	1.000	1.000	0.938

Abbreviations: BV = Bullying Victimization, AS = Academic Stress, PR = Parental Rejection , STR = Stress, ANX = Anxiety, SA = Social anxiety, OSS = Online social support, SVA = Short Video Addiction.

Step 3. Equality of Composite Means and Variances

Construct	Mean Difference	95% CI	p-value	Variance Difference	95% CI	p-value
BV	0.165	[-0.092, 0.094]	-	0.262	[-0.415 , 0.421]	**0.211**
AS	0.121	[-0.089, 0.092]	0.013	-0.071	[-0.114 , 0.108]	0.203
PR	0.054	[-0.091, 0.090]	0.272	-0.023	[-0.186 , 0.188]	**0.787**
STR	0.190	[-0.090, 0.090]	-	-0.018	[-0.141 , 0.137]	0.800
ANX	0.177	[-0.088, 0.087]	-	-0.031	[-0.138 , 0.145]	**0.702**
SA	0.094	[-0.092, 0.097]	0.052	-0.070	[-0.099 , 0.103]	0.195
OSS	0.051	[-0.091, 0.094]	0.298	0.009	[-0.130 , 0.133]	**0.878**
SVA	0.159	[-0.095, 0.084]	-	-0.026	[-0.130 , 0.111]	0.647

Abbreviations: BV = Bullying Victimization, AS = Academic Stress, PR = Parental Rejection , STR = Stress, ANX = Anxiety, SA = Social anxiety, OSS = Online social support, SVA = Short Video Addiction.
